# Advanced strategies in improving the immunotherapeutic effect of CAR‐T cell therapy

**DOI:** 10.1002/1878-0261.13621

**Published:** 2024-03-08

**Authors:** Minmin Wang, Linzi Jia, Xiangpeng Dai, Xiaoling Zhang

**Affiliations:** ^1^ Key Laboratory of Organ Regeneration and Transplantation of Ministry of Education First Hospital of Jilin University Changchun China; ^2^ National‐Local Joint Engineering Laboratory of Animal Models for Human Disease First Hospital of Jilin University Changchun China; ^3^ Department of General Medicine Shanxi Province Cancer Hospital Taiyuan China

**Keywords:** cancer vaccine, CAR‐T cell, combined therapy, CRISPR/Cas9, immunotherapy, tumor microenvironment

## Abstract

Chimeric antigen receptor (CAR‐T) cell therapy is a newly developed immunotherapy strategy and has achieved satisfactory outcomes in the treatment of hematological malignancies. However, some adverse effects related to CAR‐T cell therapy have to be resolved before it is widely used in clinics as a cancer treatment. Furthermore, the application of CAR‐T cell therapy in the treatment of solid tumors has been hampered by numerous limitations. Therefore, it is essential to explore novel strategies to improve the therapeutic effect of CAR‐T cell therapy. In this review, we summarized the recently developed strategies aimed at optimizing the generation of CAR‐T cells and improving the anti‐tumor efficiency of CAR‐T cell therapy. Furthermore, the discovery of new targets for CAR‐T cell therapy and the combined treatment strategies of CAR‐T cell therapy with chemotherapy, radiotherapy, cancer vaccines and nanomaterials are highlighted.

## Graphical abstract

The review summarizes and discusses recently developed or optimized strategies including the delivery of CAR gene, drug administration regimen, the CAR with molecular switch or logic gate control, allogeneic and super CAR‐T, double targets CAR‐T, CAR‐T targets for exploration, utilization of nano‐antibody/nanomaterials and gene‐editing, combination with radiotherapy, chemotherapy and vaccine, to improve the therapeutic effects of CAR‐T cells therapies.

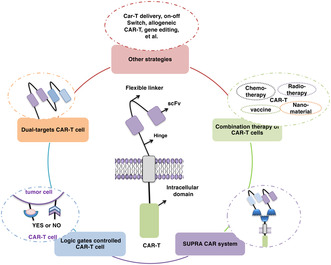

**Abbreviations**
A2AR: A2a adenosine receptor
ALPL‐1: alkaline phosphatase‐1
AML: acute myeloid leukemia
B2M: beta‐2 microglobulin
B‐ALL: B‐cell acute lymphoblastic leukemia
BTLA: B and T lymphocyte attenuator
CAF: cancer‐associated fibroblast
CAR: chimeric antigen receptor
CCA: cholangiocarcinoma
CLDN6: claudin 6
cMLV: cross‐linked multilamellar liposomal vesicles
CMV: cytomegalovirus
CRS: cytokine release syndrome
Cy: cyclophosphamide
DA‐EPOCH: dose‐adjusted etoposide, prednisone, vincristine, cyclophosphamide and doxorubicin
DC: dendritic cell
DFS: disease‐free survival
DHAP: dexamethasone, cisplatin and cytarabine
DLBCL: diffuse large B‐cell lymphoma
EphA2: erythropoietin‐producing hepatocellular carcinoma A2
FAP: fibroblast activation protein‐α
FITC: fluorescein isothiocyanate
Flu: fludarabine
GBM: glioblastoma
GMCSF: granulocyte‐macrophage colony stimulating factor
GPV: Grazoprevir Hydrate
GVHD: graft versus host disease
HER2: human epidermal growth factor receptor
HLA‐I: human leukocyte antigens class I
HVGR: host versus graft reaction
ICAR: inhibitory chimeric antigen receptors
ICE: Ifosfamide, carboplatin, and etoposide
ICIs: immune checkpoint inhibitors
IFN: interferon
IL: interleukin
IL‐13Rα2: interleukin 13 receptor subunit alpha 2
IL‐2Rβ: interleukin‐2 receptor β‐chain
IL‐6 shRNA: IL‐6 short hairpin RNA
INP: indocyanine green nanoparticles
iPSC: induced pluripotent stem cells
LNP: lipid nanoparticles
MM: multiple myeloma
mRNA: messenger RNA
MUC1: mucin‐1
NG: nanogel
NK: natural killer cells
NKG2D: natural killer group 2‐member D
NSCLC: non‐small cell lung cancer
OS: overall survival
OV: oncolytic virus
PD‐1: programmed death receptor 1
PD‐L1: programmed cell death ligand 1
PI3K: phosphatidylinositol‐3 kinase
PNE: peptide neo‐epitopes
PNP: polymer‐nanoparticle
PR: partial response
PSCA: prostate stem cell antigen
R/RMM: recurrent and/or refractory multiple myeloma
RNA‐LPX: RNA‐lipoplexes
scFv: single chain fragment variable
shRNA: short hairpin RNA
SUPRA CAR system: split, universal, and programmable CAR system
synNotch: synthetic Notch
TALEN: transcription activator‐like effector nuclease
TCL: T cell lymphomas
TCR: T cell receptor
TME: tumor microenvironment
TRAC: TCRα subunit constant
TRAIL: tumor necrosis factor‐related apoptosis‐inducing ligand
WBI: whole breast irradiation
ZFN: zinc finger nuclease
ZipCAR: universal receptor
ZipFv: scFv adaptor

## 1 Introduction

**Fig. 1:**
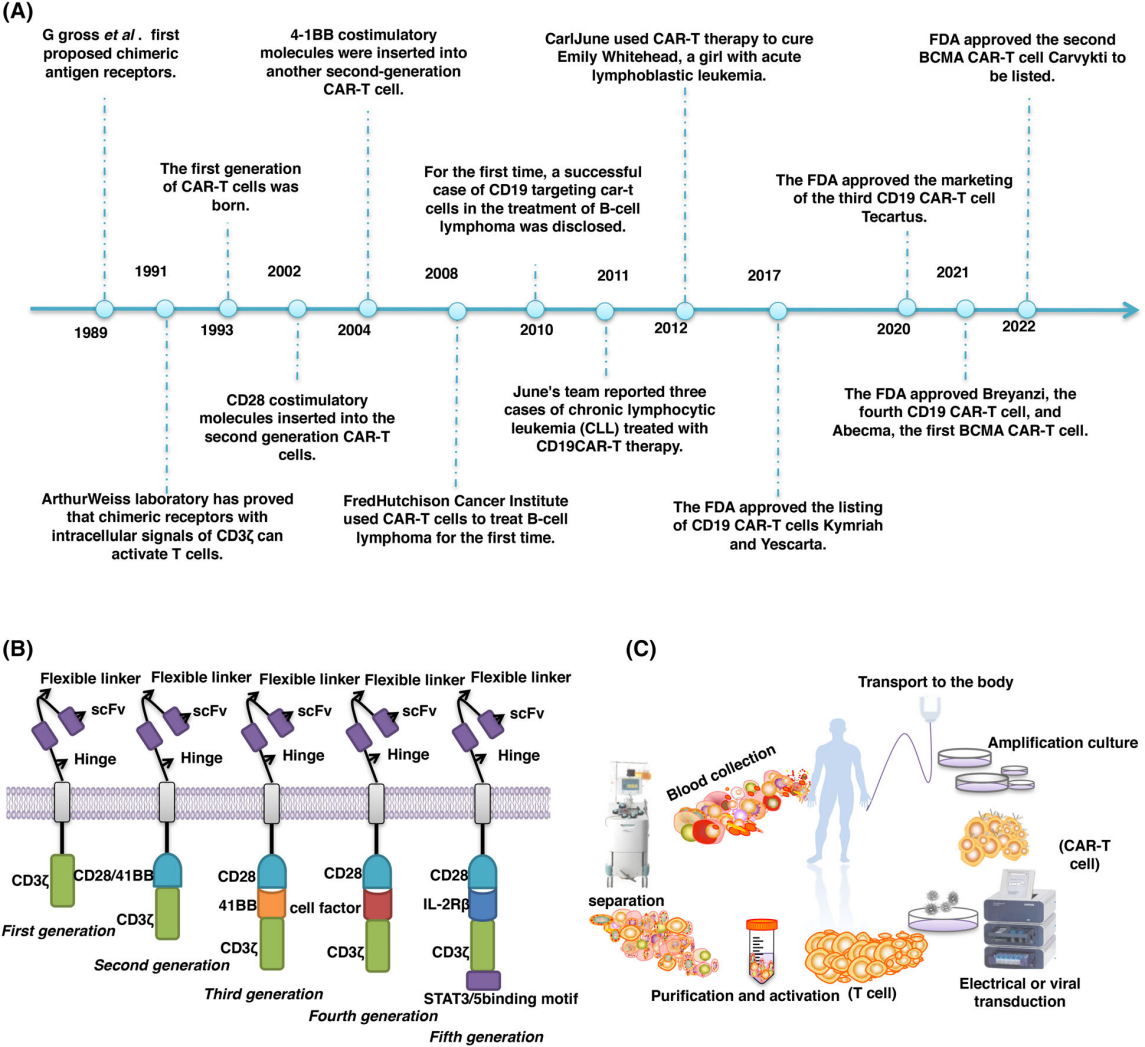
The overview of CAR‐T cells. (A) The history of the development of CAR‐T cells. (B) The first generation of CAR‐T cells relied on CD3ζ to activate T cells. The second generation added CD28 or 4‐1BB costimulatory molecules on the basis of the first generation. The third generation integrated multiple costimulatory molecules on the basis of the first generation. The fourth generation introduced some cytokines to overcome the inhibition of immune microenvironment based on the second generation. The fifth generation added domains that can activate other signal pathways. (C) Preparation of CAR‐T cells. T cells are isolated and purified from patient blood and the required CAR‐T cells are prepared by methods such as lentivirus transduction or electric transduction, and then further purified and cultured, and finally introduced into patients.

CAR‐T cells were generated through genetic modification of T cells by including the chimeric antigen receptor (CAR) to reactivate T cells to recognize the tumors which now are widely used to treat blood cancers including lymphomas, leukemia and multiple myeloma [1, 2, 3]. CAR‐T cell therapy has been developed over 30 years and has achieved promising therapeutic effects in the clinic (Fig. 1A). CAR‐T cell therapy is now considered a promising immunotherapy for human cancer. Currently, there are six CAR‐T cell therapies approved by the Federal Drug Administration (FDA) for clinical usage (Table 1). CAR‐T generally consists of four parts: the binding domain of the extracellular target antigen, the hinge domain, the transmembrane domain and one or more intracellular signal transduction domains [3, 4]. Currently, five generations of CAR‐T have been developed with different characteristics. The intracellular signal domain of the first generation of CAR‐T is CD3ζ or FcRγ with a relatively lower activation efficiency [5, 6]. Based on the first generation of CAR‐T, the second generation was produced by adding the costimulatory molecule CD28 or 4‐1BB to improve the activation efficiency of the first generation of CAR‐T [7]. The third generation of CAR‐T was also developed based on the first generation by integrating multiple signal domains, including CD3ζ‐CD28‐41BB as costimulatory molecules [8]. However, the third generation of CAR‐T did not exhibit a better activation efficiency than the second generation [9]. Furthermore, based on the second generation of CAR‐T, the fourth generation was produced by integrating some specific cytokines such as IL‐12, which not only enhanced the anticancer effect but also extended the functional time of CAR‐T cells [10]. Thereafter, the fifth generation of CAR‐T cells was generated by inserting the interleukin‐2 receptor β‐chain [interleukin (IL)‐2Rβ] between the domains of CD3ζ and CD28 and adding the transcription factors STAT3 and STAT5 at the end of CD3ζ to improve the anti‐tumor efficacy of CAR‐T cells (Fig. 1B) [11].

**Table 1:** CAR‐T cell products approved by the FDA.

Date	Target	Name	Indications
2017	CD19	KYMRIAH® (tisagenlecleucel)	1. Pediatric and young adult relapsed or refractory (r/r) B‐cell acute lymphoblastic leukemia (ALL)
			2. Adult relapsed or refractory (r/r) diffuse large B‐cell lymphoma (DLBCL)
			3. Adult relapsed or refractory (r/r) follicular lymphoma (FL)
2017	CD19	YESCARTA® (axicabtagene ciloleucel)	1. Adult patients with large B‐cell lymphoma that is refractory to first‐line chemoimmunotherapy or that relapses within 12 months of first‐line chemoimmunotherapy
			2. Adult patients with relapsed or refractory large B‐cell lymphoma after two or more lines of systemic therapy, including diffuse large B‐cell lymphoma (DLBCL) not otherwise specified, primary mediastinal large B‐cell lymphoma, high grade B‐cell lymphoma, and DLBCL arising from follicular lymphoma
			3. Adult patients with relapsed or refractory follicular lymphoma (FL) after two or more lines of systemic therapy
2020	CD19	TECARTUS® (brexucabtagene autoleucel)	1. Adult patients with relapsed or refractory mantle cell lymphoma (MCL)
			2. Adult patients with relapsed or refractory B‐cell precursor acute lymphoblastic leukemia (ALL)
2021	CD19	BREYANZI® (lisocabtagene maraleucel)	Adult patients with large B‐cell lymphoma (LBCL), including diffuse large B‐cell lymphoma (DLBCL), high‐grade B‐cell lymphoma, primary mediastinal large B‐cell lymphoma, and follicular lymphoma grade 3B who have:
			1. Refractory disease to first‐line chemoimmunotherapy or relapse within 12 months of first‐line chemoimmunotherapy
			2. Refractory disease to first‐line chemoimmunotherapy or relapse after first‐line chemoimmunotherapy and are not eligible for hematopoietic stem cell transplantation (HSCT) due to comorbidities or age
			3. Relapsed or refractory disease after two or more lines of systemic therapy
2021	BCMA	ABECMA® (idecabtagene vicleucel)	Adult patients with relapsed or refractory multiple myeloma after four or more prior lines of therapy, including an immunomodulatory agent, a proteasome inhibitor and an anti‐CD38 monoclonal antibody
2022	BCMA	CARVYKTI® (ciltacabtagene autoleucel)	Adult patients with relapsed or refractory multiple myeloma, after four or more prior lines of therapy, including a proteasome inhibitor, an immunomodulatory agent and an anti‐CD38 monoclonal antibody

Although CAR‐T cell therapy has achieved promising results in acute lymphoblastic leukemia and B cell lymphoma, many limitations still hamper the application of CAR‐T cell therapy in treating solid tumors [12, 13]. Moreover, the cell migration, tumor infiltration, selection of specific targets immune escape and cell‐targeted or non‐targeted toxicity of CAR‐T cells are important factors affecting the therapeutic effect of CAR‐T cell therapy [14, 15]. The major underlying mechanism for drug resistance of CAR‐T cells is antigen escape. First, tumor cells display high heterogeneity and have a high mutation rate, making the antigen structure also liable to changes, and ultimately the antigen will not be recognized normally [16]. Secondly, tumor cells can escape elimination by CAR‐T cells via downregulating or losing the expression of the CAR‐T cell recognition antigen [17]. Furthermore, whether CAR‐T cells can keep a high activity status over a longer period of time is also an important factor affecting their anti‐tumor role [18]. Moreover, an excessive activation of T cells or higher affinity with antigen targets could lead to the exhaustion of T cells and decrease the activity and durability of CAR‐T cells [19]. Notably, in solid tumors, the existence of immunosuppressive cells and molecules in the immunosuppressive microenvironment could be detrimental to CAR‐T cell function. For example, adenosine, a vital substance that can induce tumor immunosuppression, could hamper the activity of immune cells and affect the therapeutic effect of CAR‐T cells when it binds to its receptor A2a [20].

Therefore, it is urgent to explore novel strategies to improve CAR‐T cell therapy to achieve satisfactory anti‐tumor effects in both blood and solid tumors. Fortunately, some methods are being developed to overcome the limitations of CAR‐T cell therapy in treating some solid tumors. For instance, Priceman *et al*. [21] found that the regional intraventricular delivery of HER2‐CAR‐T cells promoted the infiltration of CAR‐T cells into the tumor microenvironment and achieved a better anti‐tumor effect. In addition, CAR‐T cells that were modified to express chemokine receptors, such as CXCR2 or CXCR1, have been proven to improve the therapeutic effect by increasing cells trafficking [22]. The researchers also found that some targeted toxicity problems can be solved by modifying the antibody affinity of CAR‐T cells [23]. Notably, Sachdeva *et al*. [24] found that the Granulocyte‐Macrophage Colony Stimulating Factor (GMCSF) is a critical protein in Cytokine Release Syndrome (CRS) and the GMCSF knockout CAR‐T cells could prevent CRS. It was also found that GMCSF knockout did not affect the anti‐tumor effect of CAR‐T cells, which proved the feasibility of generating the GMCSF knockout CAR‐T to treat CRS. Researchers have recently developed strategies to target cancer‐associated fibroblasts (CAF) in tumor microenvironments to overcome physical barriers and effectively enable CAR‐T cells to infiltrate tumor sites [25]. Furthermore, researchers developed a synapse‐tuned CAR by adding PDZ binding motifs to the internal domain of CAR to form an ‘anchor domain‘ to regulate synapses, so that the extracellular part encoded by the CAR gene can better recognize and bind with the corresponding antibodies on tumor cells to form an ‘immune synapse‘, which enables CAR‐T cells to play a better anti‐tumor role [26]. Although many strategies have been developed to improve the function of CAR‐T cells, other problems still hamper the application of CAR‐T cell therapy in treating cancer patients. Therefore, a comprehensive understanding of current strategies for improving the anti‐tumor efficiency of CAR‐T cell therapy administrated alone or in combination with other tumor treatment methods may help in prompting the broad application of CAR‐T cell therapy in combating tumors.

In this review, we summarized and discussed the recently developed strategies for optimizing CAR‐T cell generation and improving the anti‐tumor efficiency combined with other tumor treatment strategies.

## 2 Delivery strategies of CAR gene

Briefly, the first step for the generation of CAR‐T is isolating and purifying the T cells from patients. The specific receptor is then artificially expressed in these T cells that can bind to specific tumor surface antigens. Next, the genetically modified T cells are subjected to amplification *in vitro*. Finally, they are injected into the same patient to recognize and kill tumor cells (Fig. 1C) [27]. Among these processes, the successful delivery of CAR genes and the administration of CAR‐T cells are essential steps for CAR‐T therapy. Therefore, researchers have developed different strategies to improve the delivery system for CAR genes and CAR‐T cells to increase the therapeutic efficacy of CAR‐T cells.

It is well known that viral vectors are the primary delivery platforms for CAR genes. Viral vectors, such as γ retrovirus and lentivirus vectors, can effectively infect cells and stably integrate the CAR gene into the donor genome with high efficiency to achieve long‐term expression [28]. However, the package of virus particles is complicated, costly and time‐consuming, and has a high risk of infection pollution. Sometimes the cell viability is also negatively affected. Moreover, due to the lower load capability of viral vectors, they have limitations for helping to express multiple genes simultaneously and also have a higher risk of random insertion, which may induce cancer [29, 30].

Therefore, researchers have developed various non‐viral vector delivery methods, including transposon delivery. The Sleeping Beauty transposon system is the most commonly used transposon tool. Compared with virus vectors, transposon‐mediated delivery of CAR genes has multiple advantages, including being safer, having a high expression efficiency, lower cost and less immunogenicity. Moreover, transposon systems can mediate the expression of multiple genes simultaneously [31, 32, 33]. However, the use of transposon systems is frequently accompanied with low transfection efficiency and random gene integration. Therefore, transposon‐mediated CAR‐T cell therapy is still in the pre‐clinical stage and needs further investigation before clinical usage [34].

Messenger RNA (mRNA) electroporation is another delivery method. It can play its role without entering the nucleus. Therefore, the frequency of mutation insertion is low for mRNA electroporation, which is considered a safer way to transfect T cells. However, mRNA is unstable, with a shorter half‐life, preventing CAR‐T cells from continuous expression and having a durable persistence. Moreover, compared with viral delivery, mRNA electroporation has a lower transfection efficiency [35, 36, 37, 38].

In recent years, nanomaterials such as lipid nanoparticles (LNP) have been studied to deliver CAR genes. Nanomaterials have various advantages, such as low price, low toxicity and lack of integration within host genes. However, they are also have shortcomings, including low persistence caused by transient protein expression and a complicated preparation process. Further research is needed to optimize the structure and construction process of nanomaterials for efficient delivery of CAR genes [39, 40, 41].

CAR‐T cells can also be obtained by gene‐editing techniques such as TALEN and CRISPR/Cas9, which can facilitate the CAR gene to bind to any part of the genome at a fixed point. Gene editing‐mediated CAR‐T cell generation is safer and more effective. However, the low editing efficiency and high cytotoxicity limit the clinical utilization of gene editing‐mediated CAR‐T cell production [42, 43, 44]. Altogether, many of the delivery strategies that have been developed, need further optimization to deliver the CAR genes efficiently and to promote the clinical application of CAR‐T cells therapy in human disease.

## 3 Administration regimens of CAR‐T cell

Administration of CAR‐T cells usually follows the bridging therapy and clearing of lymph nodes, which can benefit the CAR‐T cell therapy by controlling disease progress and enhancing the proliferation and anti‐tumor activity of CAR‐T cells, respectively. Several administration regimens such as the specific bridge treatment, the stranguria treatment, the drug delivery mode and other treatment schemes have been developed recently. The patient's situation is an important factor affecting the selection of a suitable treatment regimen for the patient [45, 46]. Researchers have designed multiple cell delivery methods to improve the therapeutic effect of CAR‐T cells.

Currently, intravenous systemic administration is the simplest and fastest way to deliver CAR‐T cells, especially in hematological tumor patients. However, to play an ideal anti‐tumor role, a large number of CAR‐T cells are needed for intravenous systemic administration, which is not a time‐ or cost‐efficient method [47, 48]. Moreover, intravenous systemic administration is limited in treating solid tumors because the tumor microenvironment will prevent the CAR‐T cells from reaching the tumor site [49, 50]. Furthermore, the systemic transport of CAR‐T cells can easily lead to some toxicity and cause irreversible damage to the body [51, 52].

To overcome the shortcomings of the intravenous systemic administration process, the strategy of local administration was developed. Local administration can markedly increase the efficiency of trafficking and infiltration of CAR‐T cells to the tumor site, improving the therapeutic effect on solid tumors and reducing some systemic toxicities [53, 54, 55]. However, the application of local administration in patients with multiple metastatic sites is limited. Moreover, the local cytokine storm caused by local administration is harmful to patients [56]. Interestingly, a polymer‐nanoparticle (PNP) hydrogel was designed for cell delivery. The PNP hydrogel has the same properties as biological tissues, can enclose CAR‐T cells and other cytokines, and can form a temporary inflammatory niche at the injection site, which offers hope for the treatment of distant tumors and inaccessible metastatic tumors [47].

Significantly, researchers have developed the infusion strategy combined with an intensive dose to treat relapsed or refractory multiple myeloma; results showed that the administration method was safe and effective. The method exhibited sustained treatment for patients and may represent a new administration strategy [57]. Direct generation of CAR‐T cells *in vivo* by viral vectors and nanomaterials is another option to address CAR‐T cell delivery which does not need cell administration [58, 59]. For example, Rurik *et al*. [59] recently used lipid nanoparticles to deliver mRNA and produced anti‐fibrotic CAR‐T cells *in vivo*, which could restore heart function and enable *in vivo* generation of CAR‐T cells. Due to the heterogeneity and complexity of tumors, optimizing the currently used drug delivery strategies and exploring relevant drug delivery mechanisms and novel drug delivery systems to achieve better anti‐tumor effects are still necessary.

## 4 Dual‐target CAR‐T cells

The high heterogeneity and immune escape are the main characteristics of cancer cells, enabling them to escape elimination by the immune system. CAR‐T cells have the function to activate T cells, and the activated T cells can then recognize escaped tumor cells and kill them [6]. To enhance the therapeutic effect of CAR‐T cell therapy, researchers have modified the structure of CAR so that CAR‐T cells can target multiple antigens simultaneously. For instance, using double CAR constructs connecting two scFv (TanCAR) with the same CAR or directly adding different CAR‐T cells in the treatment could achieve simultaneous multi‐target therapy and inhibit the immune escape caused by antigen loss or weakened expression of antigen [60, 61, 62].

**Fig. 2:**
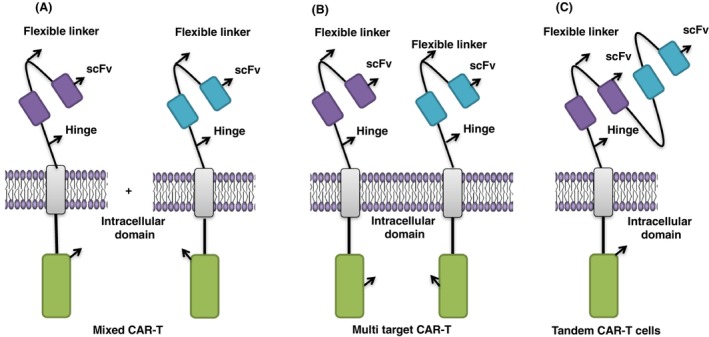
Dual‐target CAR‐T cells. (A) Mixed CAR‐T. CAR‐T cells with two different targets were directly added to the treatment process. (B) Multi‐target CAR‐T. Two CAR targets are expressed on the same T cell simultaneously to fight tumor cells. (C) Tandem CAR‐T cells. Two targets are connected in series in the extracellular domain of T cells to recognize different antigens.

Two kinds of CAR‐T cells with different antigen targets were obtained through transduction with two different vectors, and these two kinds of CAR‐T cells were used for double‐target therapy (Fig. 2A). CAR‐T cells with mucin‐1 (MUC1) and prostate stem cell antigen (PSCA) cannot eliminate tumor cells but the combined therapy showed a better anti‐tumor effect than single CAR‐T cell therapy [63]. In addition, the targets of CAR‐T cells can be on the surface of tumor cells as well as on the tumor‐related matrix. For example, Kakarla *et al*. [64] found the CAR‐T cells targeting fibroblast activation protein α (FAP) in the tumor‐related matrix in combination with CAR‐T cells targeting the erythropoietin‐producing hepatocellular carcinoma A2 (EphA2) antigen could significantly increase the anti‐tumor effect and prolong the survival time of tumor‐bearing mice. Moreover, cocktail immunotherapy was developed using two different CAR‐T cells for dual‐target therapy (other CAR‐T sequential treatments). A patient with advanced cholangiocarcinoma (CCA) successively received EGFR and CD133 CAR‐T cells therapy and showed a partial response (PR) continuously, which proved the feasibility of the cocktail immunotherapy [65]. Another clinical experiment showed that CAR‐T cell cocktail therapy of CD19/CD22 for B‐cell malignant tumors was effective and safe for patients [66].

The other dual‐target CAR‐T cell was generated by expressing two CAR targets on the same T cell (Fig. 2B) [67]. The initial treatment with a single CAR‐T cell achieved good results but later the patient, who had acute myeloid leukemia (AML), showed recurrence of the disease. Therefore, researchers developed dual‐target CAR‐T cell therapy by targeting two different antigens, CD123 and CD33, and achieved a good therapeutic effect in treating AML, proving the effectiveness of this therapy [68].

The tandem chimeric antigen receptor (TanCAR) is another dual‐target CAR‐T cell, connecting two targets in series in the extracellular domain of T cells (Fig. 2C). The dual‐specific CAR‐T cells, formed via connecting BCMA and CD19scFv through CAR, showed extraordinary safety and effectiveness in treating multiple myeloma (MM) [69]. In addition, CAR‐T cells with dual specificity for BCMA/CS1 and the BCMA‐OR‐CD38Tan CAR‐T cells have achieved satisfactory therapeutic effects in treating multiple myeloma (MM) [70, 71]. The TanCAR T cells, constructed by including interleukin 13 receptor α2 (IL‐13Rα2) and EphA2, can not only eliminate giant tumors but also play the potential role in preventing antigen escape [72]. Notably, Hegde *et al*. proved that TanCAR T cells showed the best anti‐tumor ability by targeting HER2 and IL‐13α2 in the glioblastoma model, followed by targeted CAR‐T cells and finally mixed CAR‐T cells [61, 73].

## 5 Logic gates‐controlled CAR‐T cell

The logic gate‐controlled CAR‐T cells were designed to overcome problems including antigen escape. This kind of CAR usually needs at least two or more antigen binding sites to function. The main logic gates used for the logic gates controlled CAR‐T cells include ‘AND gate’, ‘OR gate’ and ‘NOT gate’ [74, 75, 76].

### 5.1 AND logic gate

**Fig. 3:**
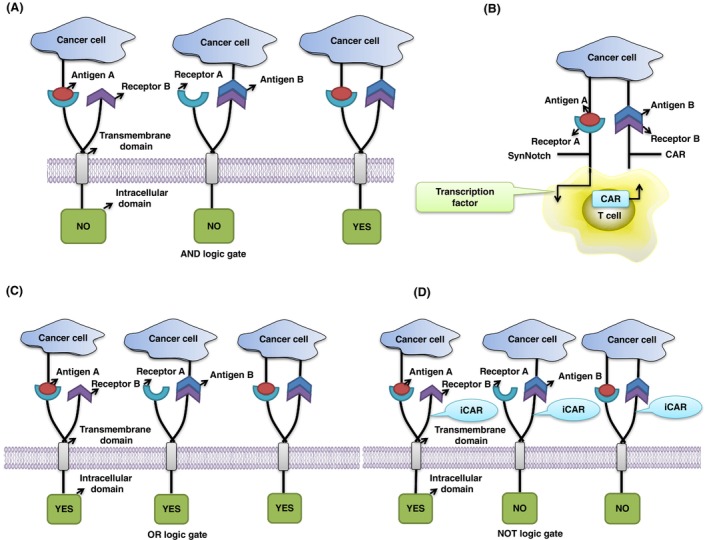
Logic gate‐controlled CAR‐T. (A) AND logic gate. Only when all the targets are bound by tumor cells can T cells be activated to play an anti‐tumor role. (B) Synthetic Notch (SynNotch)‐CAR‐T cells. When the antigen on tumor binds to a CAR‐T cell receptor, it activates the transcription and expression of another antigen receptor on T cells; when it binds to tumor cells again, it activates T cells and exerts an anti‐tumor effect. (C) OR logic gate. Upon binding the target, T cells can be activated kill the tumor. (D) NOT logic gate. In addition to the receptor signal activated by T cells, there are also inhibitory chimeric antigen receptors (iCAR). When iCAR is recognized, it hinders the activation of T cells to protect the normal cells from T cell‐mediated damage.

The AND logic‐gated CAR‐T cell has two or more antigen targets, each of them containing a stimulus site. T cells can be fully activated and exert their anti‐tumor effect only when both the stimulus sites are activated. That is to say, only when these antigens exist simultaneously on one tumor cell can they be recognized and the CAR‐T cells activated to play their anti‐tumor role [76]. Because AND logic‐gated CAR‐T cells can only function when multiple antigens are present on the same tumor cell, the AND logic‐gated CAR‐T cell can dramatically reduce the non‐specific targeting and non‐tumor toxicity (Fig. 3A) [77, 78]. Based on AND logic‐gated CAR‐T cells, the CAR‐T cells with synthetic Notch (synNotch) were designed. After the synNotch receptor binds to a specific antigen on the tumor cell, it can release transcription factors inside the T cells and activate another CAR‐T cell target. Only when the tumor cells are combined with both targets can the T cells be activated and function (Fig. 3B) [79, 80, 81]. Choe *et al*. also measured the effect of the synNotch CAR‐T cells in glioblastoma. They found the CAR‐T cells have a longer survival time and higher accuracy in targeting the corresponding tumor cells [82]. Other researchers have also developed an AND logic‐gated CAR‐T cell utilizing AxlscFv as a synNotch receptor, which showed an excellent therapeutic effect on colon cancer, pancreatic cancer and breast cancer cells expressing Axl receptor tyrosine kinase [83]. Moreover, tumor‐specific synNotch receptors also exhibit advantages in locating CAR‐T cells in the tumor microenvironment. The synNotch receptor designed by Allen *et al*. [84] can locally induce the production of inflammatory cytokine IL‐2, enhance the infiltration of CAR‐T cells, and clear immune rejection tumors.

### 5.2 OR logic gate

OR logic‐gated CAR also involves multiple antigen receptors. As long as one antigen on tumor cells binds to the target on CAR‐T cells, the anti‐tumor effect of T cells will be activated. The OR logic gate control can help to reduce antigen escape occurrence caused by tumor heterogeneity (Fig. 3C) [85].

It was reported that the synthesized CAR‐T cells with dual targets of HER2 and IL‐13Rα2 showed more potent anti‐tumor activity in glioblastoma (GBM) compared with CAR‐T cells with single target [73]. Ruella *et al*. designed the OR logic‐gated CAR‐T cells with CD19 and CD123 targets, whose effect was tested in the treatment of relapsed/refractory B‐cell acute lymphoblastic leukemia (B‐ALL). The results indicated that the OR logic‐gated CAR‐T cells exhibited excellent therapeutic effects in preventing antigen loss and disease recurrence [86]. However, CAR‐T cells with dual targets still have some limitations in their anti‐tumor function due to the high tumor heterogeneity. Therefore, researchers tried to construct the CAR‐T cells with three targets or logic gates by simultaneously targeting human epidermal growth factor receptor 2 (HER2), IL‐13 receptor subunit alpha (IL‐13Rα2) and EphA2. Notably, the CAR‐T cells with three antigen targets show more robust resistance to the tumor heterogeneity in glioblastoma mouse model. These cells can capture more tumor cells to achieve better anti‐tumor effect [85, 87].

### 5.3 NOT logic gate [inhibitory chimeric antigen receptor (iCAR)]

NOT logic gates typically include two kinds of recognition antigens. One antigen has a T cell activation signal and the other antigen represents the inhibitory signal to block T cell activation [88]. Therefore, the tumor cells with one activating antigen can activate T cells. However, if the two antigens exist simultaneously or if only an inhibitory antigen exists, T cells cannot be activated due to the dominant inhibitory signal. Because the inhibitory CAR antigen is commonly expressed on normal cells, the NOT logic gate can protect normal cells from T cell‐mediated damage (Fig. 3D) [89]. Fedoroff *et al*. generated the inhibitory chimeric antigen receptors (iCAR) by combining the signal domains of immunosuppressant receptors PD‐1 and CTLA‐4 with the antigen recognition domain expressed on normal cells, which was ultimately used to construct the non‐logic gate CAR‐T cells. The iCAR system could avoid some side effects such as cytokine release and cytotoxicity caused by receptor activation of T cells and other problems caused by the lack of specificity of CAR‐T cell targets [89].

In summary, the CAR‐T cells with biological logic gates could partially reduce the antigen escape and non‐tumor targeting cytotoxicity, improving the therapeutic effect and safety of modified CAR‐T cells in the treatment of cancers.

## 6 SUPRA CAR system

Although CAR‐T cell therapy was considered a promising therapeutic strategy for blood cancer patients, there are still some limitations concerning its safety, effectiveness and specificity for the treatment of solid tumors [90, 91]. Moreover, because CAR‐T cells are customized individually and need a longer production cycle and higher cost, their wide application in treating various tumors is limited. Therefore, the design of universal and flexible CAR‐T cells should be investigating in the future [92].

**Fig. 4:**
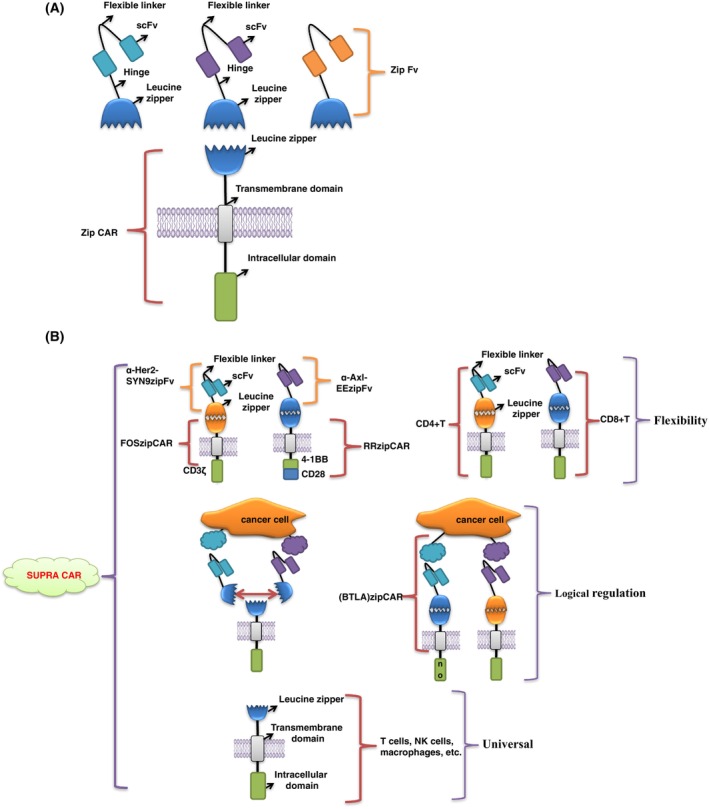
Split, universal and programmable (SUPRA) CAR‐T cells. (A) SUPRA CAR‐T cells. These consist of two independent parts, the universal receptor (ZipCAR) expressed in T cells and the scFv adaptor (ZipCAR) specifically targeting tumor antigen. ZipCAR is composed of an intracellular signal and extracellular leucine zipper, and ZipFv is composed of scFv and leucine zipper targeting tumor antigen. When ZipCAR and ZipFv are combined by homologous leucine zipper, they can play the same role as traditional CAR‐T cells. (B) The SUPRA CAR system can flexibly and independently control different signal regions to modulate different types of T cells and subsequently achieve flexibility, versatility and logical regulation of T cell function.

In line with this notion, a split, universal and programmable (SUPRA) CAR system was produced to improve the flexibility, safety and anti‐tumor effect of CAR‐T cells. The SUPRA CAR system consists of two parts: the universal receptor (ZipCAR) located on T cells, and the scFv adaptor (ZipFv) specifically targeting tumor antigens. The ZipCAR comprises an intracellular signal domain and an extracellular leucine zipper. ZipFv adaptor is composed of extracellular specific scFv and leucine zipper. Homologous leucine zippers on ZipCAR and ZipFv can be connected to form normal CAR‐T cells, which can play the same role as traditional CAR‐T cells (Fig. 4A). CAR‐T cells of the SUPRACAR system have shown satisfactory therapeutic effects in the *in vivo* experiments on the breast cancer and leukemia xenograft mice models [93].

As we know, traditional CAR‐T cells are composed of antigen‐binding scFv fixed outside T cells and signal domains fixed inside T cells, which markedly limits the flexibility of CAR‐T cells [94]. However, in the SUPRA CAR system, the ZipCAR and ZipFv are separated, and the intracellular signal domain of ZipCAR can be modified separately. Researchers have designed various orthogonal SUPRA CAR to control different signal pathways independently. Examples include FOSZipCAR (combined with α‐Her2‐SYN9ZipFv) to control the CD3ζ region, and RRZipCAR (combined with α‐Axl‐EEZipFv) to manage the activation domains of 4‐1BB and CD28, which could better regulate the T cell activation and achieve a better therapeutic effect on tumors. In addition, orthogonal SUPRA CAR can also be used to control the activation and function of different T cells. For example, different ZipFv can be used to regulate the function of CD4^+^ T cells (to help secrete cytokines) and CD8^+^ T cells (to kill cancer cells directly), thus improving the release of some anti‐tumor cytokines, which help CAR‐T cells to achieve better anti‐tumor outcomes [93, 95].

In addition, the CAR‐T cells with a single target antigen can easily lead to drug resistance of tumor cells. However, when the CD19 and CD22 dual‐target CAR‐T cells were utilized to treat B‐cell acute lymphoblastic leukemia, the tumor cells were still able to develop resistance to CAR‐T cell therapy by downregulating the expression of CD19 and CD22 antigens [16]. If we continue to add new targets to fight against tumor cells on this basis, we need to retransform T cells, a time‐consuming and expensive process. In the SUPRA CAR system, because ZipFv and ZipCAR are separated, we can target different antigens only by adding different ZipFv, which dramatically improves the treatment efficiency of CAR‐T cells. This is similar to the OR logic gate of traditional CAR‐T cells [93].

Notably, the nonspecific antigens expressed on both tumor and normal cells are another challenge for CAR‐T cell therapy. The NOT logic‐gated CAR‐T cells with an antigen target only expressed on the normal cells, can increase specificity. When the target is recognized, the activation of T cells will be inhibited, and the normal cells will be protected from T cell‐mediated killing [89]. In the SUPRA CAR system, researchers also designed CAR‐T cells similar to non‐logic‐controlled CAR‐T cells to protect normal cells from being mistakenly identified. Notably, two ZipFv were added to the SUPRA CAR system – one was expressed in normal cells and the other was a nonspecific target that could target tumors. When the antigen targets correlated to the two ZipFv expressed on cells simultaneously, the two ZipFv would be combined by a leucine zipper, but not with the ZipCAR, which blocks the activation of T cells to protect the normal cells from damage caused by CAR‐T cells. However, if only one antigen is expressed on the cell, the ZipFv can bind to both the specific antigen and the ZipCAR to activate T cells [93].

Furthermore, researchers have designed another non‐logic gate‐controlled CAR‐T cell which is more similar to the traditional CAR‐T cells. The B and T lymphocyte attenuator (BTLA) ZipCAR was produced using the co‐inhibitory receptor; when (BTLA) ZipCAR was activated in the constructed non‐logic gate CD4^+^ T cells, it could inhibit the IFN‐γ secretion of CD4^+^ T cells. In natural killer (NK) cells, the constructed non‐logic gate BTLA can significantly reduce the killing efficiency of NK cells. Therefore, the ‘NOT gate’ of SUPRA CAR could be used to control the killing accuracy of immune cells. In addition, other logic gates such as AND logic gates can also be easily implemented in the SUPRA CAR system [96]. Therefore, the SUPRA CAR system can be a platform to mediate the tumor‐killing effect for different kinds of immune cells (Fig. 4B).

Moreover, the biotin‐bound CAR‐T cells could be constructed through the CD2‐CD28 and 3‐4BB‐CD1ζ signal domains. When biotinylated antibodies, such as CD19 and CD20, are injected into patients, the antibodies can bind to the tumor cells and biotin‐bound CAR‐T cells can bind to these biotinylated antibodies on tumor cells to activate T cells [97]. Besides biotin, other molecules, such as fluorescein isothiocyanate (FITC) and peptide neo‐epitopes (PNE), can be used to construct these kinds of universal CAR‐T cells [97, 98, 99]. Benefitting from the concept of CAR‐T cell therapy, other immune cells, such as NK cells, macrophages and other T cell subtypes, were also used to construct CAR‐M or CAR‐NK cells [100, 101].

## 7 ON/OFF switches in CAR‐T cells

The cytokine release syndrome (CRS) is one of the adverse symptoms of CAR‐T cell treatment *in vivo*, leading to high fever and rapid decrease in blood pressure in patients. Therefore, researchers have designed molecular switches to control CAR‐T cells remotely, improving their flexibility for control and safety [102, 103, 104]. For example, loading CAR‐T cells with photothermal sensitive switches can trigger the expression of the CAR gene only when the temperature is raised to a particular level. Preclinical studies indicated this approach has higher effectiveness and safety [105]. The combination of ultrasound with CAR‐T cells to create a switch to regulate CAR‐T cells was also developed and tested [106]. In addition, a switch for ‘hunger therapy’ has been designed to modify T cells with specific nutrients whose alteration will regulate the survival of CAR‐T cells [107]. Importantly, a series of small molecule compounds, such as dasatinib and resveratrol, have been designed to regulate CAR‐T activity [108, 109]. Recently, a new molecular switch, SNIPCAR, was developed based on a protease system which utilizes FDA‐approved small molecules NS3p inhibitor Grazoprevir Hydrate (GPV) to regulate CAR‐T cell activity. The CAR‐T cells can function normally in the presence of these drugs and will not work without drugs. Compared with traditional CAR‐T cells, those CAR‐T cells with ON/OFF switches showed higher safety and effectiveness [110]. Therefore, exploration of novel CAR‐T switch systems is of great significance for accurate regulation of CAR‐T cell function in cancer therapy.

## 8 Allogeneic CAR‐T cells

The autologous CAR‐T therapy can avoid clearance by the patient's immune system and has a satisfactory therapeutic effect. However, it also has limitations, such as long customization times, high cost and the difficulty getting a sufficient number of T cells for CAR‐T cell preparation in some patients. Therefore, the allogeneic CAR‐T cells were developed to overcome the shortcomings of autologous CAR‐T therapy.

A large number of T cells obtained from healthy donors could be used to generate CAR‐T cells to treat multiple patients. However, the imported allogeneic T cells may attack the host organs, leading to graft versus host disease (GVHD). In addition, allogeneic T cells may be recognized and killed by the host's immune cells, thereby reducing their anti‐tumor effect and leading to immune rejection (host versus graft reaction, HVGR) [111, 112, 113]. Fortunately, researchers can use the gene‐editing techniques to transform CAR‐T cells to solve these problems. The TALEN gene‐editing technique was reported to prepare allogeneic CAR‐T cells lacking TCRαβ and MHC‐1 and expressing NK cell inhibitor HLA‐E so that they could escape the attacks of host NK cells and allogeneic T cells and have extended durability and higher anti‐tumor activity [114]. Furthermore, CRISPR/Cas9 technology was used to eliminate the rejection of allogeneic CAR‐T cells by knocking out the two genes of *TRAC* (TCRα subunit constant) and *B2M* (beta‐2 microglobulin) and the three genes *TRAC*, *B2M* and *PD‐1* (programmed death receptor 1) as well as *TCR* (T cell receptor) and *HLA‐I* (human leukocyte antigen class I) [115, 116, 117]. Furthermore, short hairpin RNA (shRNA) could also be used to reduce T cell receptor (TCR) expression at the transcriptional level, and some chemotherapy drugs were used to suppress the host's immune system to avoid immune rejection [118, 119]. Although allogeneic CAR‐T cell therapy is considered a promising method, numerous problems still need to be solved before the allogeneic CAR‐T cells therapy can completely replace autologous CAR‐T cells therapy. Recently, Jing *et al*. used human‐induced pluripotent stem cells (iPSCs) to produce TCRαβ T cells that are similar to those in peripheral blood. Similar to the natural T cells, the iPS‐derived T cells could be used to generate the CAR‐T (CAR‐iPS‐T) cells, which showed vigorous anti‐tumor activity in the mouse model of B‐cell lymphoma. This result suggested that the iPS‐derived T cells could be a new T cell source to form the universal CAR‐T cells [120].

## 9 Discovery of specific targets for CAR‐T cell therapy

Given that targets of CAR‐T cells which expressed on the tumor cells are essential to eliminate tumor cells accurately, the discovery of specific targets will promote the efficacy of CAR‐T cell therapy in cancer treatment without affecting the normal tissues.

The protein level of OR2H1 is higher in colon cancer and gallbladder cancer tissue but are lower in the testis. Therefore, the OR2H1CAR‐T cells might have higher specificity to kill OR2H1‐positive tumor cells selectively without affecting normal cells [121]. Because alkaline phosphatase‐1 (ALPL‐1) is highly specific in both primary and metastatic osteosarcoma, CAR‐T cell therapy targeting ALPL‐1 demonstrated higher effectiveness in treating cancer in preclinical trials [122]. Due to the high heterogeneity of tumor cells and lack of specific targets, CAR‐T cell therapy has not yet achieved a satisfactory therapeutic effect in treating acute myeloid leukemia (AML). Recently, Gottschlich *et al*. [1] found CSF1R and CD86 targets by analyzing RNA sequencing data and constructed the correlated CAR‐T cells and demonstrated strong therapeutic effects in both *in vitro* and *in vivo* models. Moreover, in a clinical study (ChiCTR210048888) of multiple myeloma (MM), GPRC5D was found to be a new alternative target, with GPRC5D CAR‐T cells playing a prominent role in MM patients not responding to BCMA CAR‐T cell therapy or patients with recurrence after treatment [123].

CAR‐T cell therapy and engineered TCR therapy are two cell therapies aiming to pursue successful cancer treatment by modifying the artificial or natural receptors. Moreover, CAR‐T cell can be considered an engineered TCR‐T cell. In contrast to CAR‐T cells, which will generate an artificial receptor on T cell, TCR will modify the natural receptors on T cells to promote their recognition and binding with tumor cells in a more efficient way. Furthermore, the generation of CAR‐T cells is limited by the number of surface antigens of tumors. TCR‐T cells, which can target the antigens inside tumors, may play a wide role in the treatment of solid tumors in the future [124]. Due to their difference, TCR‐T cell therapy is a separate topic, and we will not discuss them in detail here. In line with this notion, whether CAR T cell therapy and TCR‐T cell therapy can complement each other or their combination therapy could enhance anti‐tumor effect needs further in‐depth investigation.

## 10 Nanobodies in CAR‐T cell therapy

Nanobodies were first discovered in camels in 1993 [125]. Now they are widely used in many research fields due to their small size, simple structure, high stability and high affinity [126, 127, 128, 129]. In CAR‐T cells, nanobodies exhibit some advantages over scFv of traditional monoclonal antibodies. A nanobody is easier to be humanized due to low immunogenicity and a sequence similar to the heavy chain variable region of humans [130]. Moreover, researchers have found that in TanCAR formed by two different tandem antibodies, nanobodies exhibit better therapeutic effects due to their lack of interference between heavy and light chains [61]. However, CAR‐T cells based on nanobodies generally do not aggregate on the CAR surface, resulting in CAR‐T depletion and enabling CAR‐T cells to function more sustainably [131]. Notably, the ciltacabtagene autoleucel (*cilta‐cel*) CAR‐T, an FDA‐approved nano antibody product, was used to treat recurrent and/or refractory multiple myeloma (R/RMM) in adults by targeting mature B cell antigens (BCMA) through the recognition of two distinct epitopes of BCMA simultaneously using its dual binding domain. Therefore, one *cilta‐cel* with dual binding domain could recognize and bind with two proteins with *cilta‐cel* targets and promote the formation of a strong immune synapse [132].

In addition, Mo *et al*. constructed nanobody‐based CD105 CAR‐T cells and tested their anti‐tumor functions in hepatocellular carcinoma. The results showed that CD105 CAR‐T cells could induce T cell activity and production of pro‐inflammatory cytokines and carry out tumor‐killing roles [133]. Recently, Li *et al*. [134] constructed a CAR‐T cell based on the nanobody B7‐H3 and demonstrated that the CAR‐T cells have a practical tumor‐fighting effect against large solid tumors in female mice models.

Moreover, the nanobody‐based CD19 CAR‐T, CD20 CAR‐T and bispecific CAR‐T cells demonstrated high potential to kill Burkitt lymphoma tumor cells and patient‐derived tumor cells *in vitro*, further confirming the effectiveness of nanobody‐based CAR‐T cell therapy [135]. Notably, researchers generated nanobody‐based AML‐specific antigen CA70 CAR‐T cells which showed a better therapeutic effect than CD70 CAR‐T cells in AML. The finding implied that the expression of CD70 induced by the drug in combination with CA70 CAR‐T cells could further enhance their therapeutic function [136]. Importantly, nanobody‐based dual‐target therapies have also exhibited enhanced tumor‐killing effects. Xia *et al*. [137] demonstrated that the nanobody‐based CD30‐CD5‐CAR‐T cells exhibited superior anti‐tumor effects against T cell lymphoma (TCL) in both *in vivo* and *in vitro* experiments, suggesting that nanobody‐based CAR‐T cells could dramatically improve their anti‐tumor efficiency compared with conventional scFv‐derived CAR‐T cells.

In future, nanobody‐based CAR‐T cells will attract more attention, and their administration alone or in combination with other CAR‐T cells therapies might achieve better therapeutic effect.

## 11 Gene‐editing technology in CAR‐T cell therapy

Currently, gene‐editing techniques such as transposons, zinc finger nucleases (ZFN), transcription activator‐like effector nuclease (TALEN) and CRISPR/Cas9 not only play important roles in facilitating CAR gene delivery but also improve CAR‐T cell trafficking and persistence [116, 138, 139]. For example, universal CAR‐T cell depletion of TCRαβ and HLA‐ABC by TALEN‐mediated gene‐editing showed enhanced persistence and anti‐tumor activity [114]. It is well known that PD‐1/PD‐L1 is a key regulator of T cell function, and PD‐1/PD‐L1 blockage showed a positive anti‐tumor effect; therefore, the anti‐tumor efficacy of the CAR‐T cell depletion of PD‐1 by the CRISPR/Cas9 gene‐editing technique was significantly improved [140]. In addition, CRISPR/Cas9 technology can enhance T‐cell function by disrupting the expression of some co‐inhibitory molecules such as CTLA‐4 and Tim‐3 [141]. Furthermore, CRISPR/Cas9 technology was used to mediate the insertion of the CAR gene sequence into the genome of T cells, which could effectively overcome some problems including the random insertion, and stabilize the integration of CAR genes with T cell genome [142]. Moreover, CRISPR/Cas9 technology was used to target a CD19‐specific CAR to TCRα subunit constant (TRAC) sites to achieve the even expression of CAR in human peripheral blood T cells and to enhance the CAR‐T cell function examined in an acute lymphoblastic leukemia mouse model [142, 143]. Gene‐editing techniques can also be used to achieve gene knockout to resist isomeric CAR‐T cell GVHD and HVGA, as well as to break down some inhibitory molecules such as the TLA‐4 and Tim‐3 to improve T cell function, as we discussed in the section of Allogeneic CAR‐T cell. It is believed that in the near future the combination of gene‐editing techniques and CAR‐T therapy will shed light on the development of novel CAR‐T cells with strengthened anti‐tumor function.

## 12 Combined therapeutic strategy for CAR‐T cell therapy

**Fig. 5:**
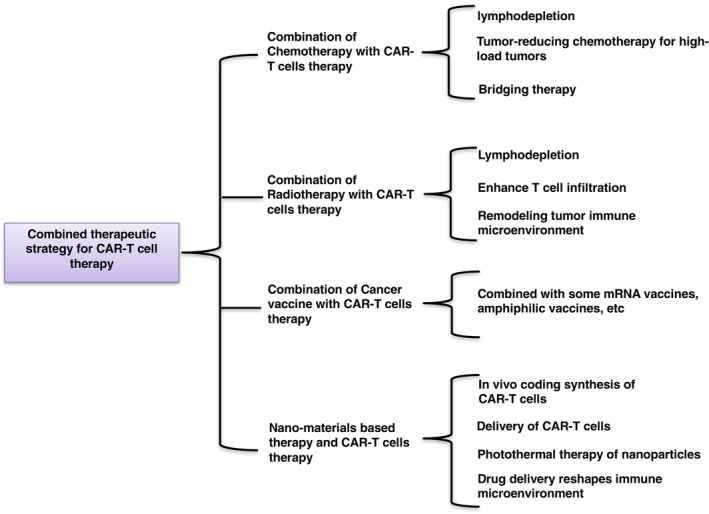
Summary of combined application of CAR‐T cells therapy with radiotherapy, chemotherapy, vaccine and nano‐materials.

It is well known that combined therapy can usually achieve better therapeutic outcomes than single therapy in treating human diseases, including cancers [144, 145, 146]. The combined therapy can not only reduce drug resistance frequently occurring during cancer treatment but also reduce toxicity by decreasing the dose of a single drug, resulting in better therapeutic effects and lower frequency of side effects [147, 148]. Therefore, to achieve better treatment outcomes, novel therapeutic strategies were developed in combination with CAR‐T cell therapy to combat cancers. Below, the combination therapies of CAR‐T cells with chemotherapy, radiotherapy, vaccines and nanomedicine are discussed in detail (Fig. 5).

### 12.1 Combination of chemotherapy with CAR‐T cell therapy

Currently, chemotherapy is the most commonly used method for the treatment of malignant tumors, and CAR‐T cell therapy is one of the most promising immunotherapies for cancer patients in recent years.

Although the anti‐CD19 CAR‐T cell therapy has achieved remarkable results in the treatment of refractory/recurrent (R/R) diffuse large B‐cell lymphoma (DLBCL), the therapeutic effect for the treatment of larger tumors is far from satisfactory. Notably, a clinical trial showed that 25 patients in the combined group were treated with different chemotherapy regimens using dexamethasone, cisplatin and cytarabine (DHAP) with ifosfamide, carboplatin and etoposide (ICE), dose‐adjusted etoposide, prednisone, vincristine, cyclophosphamide and doxorubicin (DA‐EPOCH), to pretreat DLBCL tumors, followed by treatment with CAR‐T cells. The results showed that the combined group had significantly higher disease‐free survival (DFS) and overall survival (OS) compared with the chemotherapy group, providing an effective treatment strategy for patients with a high load of DLBCLR/R tumor [149, 150]. Importantly, the two commonly used chemotherapy drugs, fludarabine and cyclophosphamide, were reported to cause short‐term decrease of lymphocytes in mice and humans, which will provide a suitable environment for the survival and proliferation of CAR‐T cells *in vivo* [151, 152]. Therefore, it may be essential to clear the lymphocyte with chemotherapy before CAR‐T cell therapy in cancer patients. Turtle *et al*. found that the cyclophosphamide (Cy) and fludarabine (Flu) treatment dramatically improved the therapeutic effect of CAR‐T cells therapy on relapsed/refractory B cell NHL cancer [95, 153].

At present, it has been proposed that there are several mechanisms underlying the improved function of CAR‐T cell therapy in combination with chemotherapy, which include the tumor reduction caused by the treatment of chemotherapy before CAR‐T cell therapy, the bridging therapy, which is used to control the progress of the disease by some drug during T‐cell collection, and final CAR‐T cell infusion therapy. At the same time, lymphocyte clearance achieved by chemotherapy provides a suitable environment for the function of CAR‐T cells [154, 155].

### 12.2 Combination of radiotherapy with CAR‐T cell therapy

Radiotherapy can inhibit tumor growth and increase the anti‐tumor function of immune cells [156]. Therefore, the combination of radiotherapy and immunotherapy may further promote immunotherapy [157, 158].

Previous studies have shown that lymphocyte removal before T cell implantation and expansion can increase the therapeutic effect of CAR‐T cell therapy [159, 160]. Murty *et al*. found that the mice with glioblastoma (GBM) receiving whole breast irradiation (WBI) followed by GD2‐CAR‐T cell therapy showed improved anti‐tumor response and a prolonged survival time. Furthermore, memory T cells with specific antigens induced by the combined treatment of WBI and GD2‐CAR‐T cells could inhibit the growth of the replanted tumor cells. Mechanistically, the T cells of irradiated mice can infiltrate quickly and further expand in the tumor microenvironment, which implies that CAR‐T cells might play an important role in the tumor infiltration of immune cells [161]. In addition, Qu *et al*. found that the strategy of radiotherapy followed by targeting CD19, CD20 or CD22 CAR‐T cell treatment is a safer and more effective scheme in treating R/RDLBCL with high tumor load because the radiotherapy may play a role in supplying the CAR‐T cells and resisting CRS by decreasing the local tumor IL‐6 [162, 163].

The high heterogeneity of tumor cells is a major cause for antigen escape during CAR‐T cell therapy, and various tumor cells will express different tumor‐associated antigens, which can affect the therapeutic effect of CAR‐T cell therapy. It was reported that low‐dose radiation combined with CAR‐T cell therapy could achieve better therapeutic effects in pancreatic cancer models [164]. It was also shown that in heterogeneous tumors, after radiotherapy and CAR‐T cell therapy, the positive tumor cells expressing the corresponding antigen of CAR‐T cells can express higher TRAIL (tumor necrosis factor‐related apoptosis‐inducing ligand). This can further cause significant apoptosis of tumor cells that do not express the corresponding antigen and reduce the antigen escape [164]. In line with this notion, radiotherapy was found to promote the release of tumor‐associated antigens and some signaling molecules, which help to build a microenvironment beneficial for tumor regression and ultimately increase the penetration ability and antitumor efficacy of CAR‐T cells [165, 166].

Effective trafficking of CAR‐T cells to the tumor microenvironment plays an essential role in cancer treatment. The transport of CAR‐T cells into the tumor microenvironment is dynamic, involving many different processes, such as cell rolling and adhesion, some extravasations and chemotaxis, and is also influenced by chemokines [167, 168]. Dovedi *et al*. found that radiotherapy can enhance T cell infiltration into tumors by increasing cell adhesion, rolling, extravasation, chemotaxis release and reshaping the blood vessels inside the tumor [162, 169, 170, 171]. Furthermore, radiation can significantly increase the expression of natural killer group 2‐member D (NKG2D) ligand in glioblastoma [172]. Weiss *et al*. found that radiation could promote the infiltration of NKG2D CAR‐T cells into tumor cells and increase interferon gamma (IFN‐γ) secretion *in vivo* and *in vitro*. Therefore, radiotherapy plays an essential role in enhancing the efficiency of CAR‐T cell therapy partially through regulating lymphocyte removal, antigen release, T cell infiltration and trafficking [173]. However, the combined therapeutic efficiency of radiotherapy and CAR‐T cell therapy is affected by the treatment dose, immune microenvironment and tumor type, which warrants further investigation to understand thoroughly the underlying mechanism for the improvement of combined therapy of radiotherapy and CAR‐T cell therapy [174].

### 12.3 Combination of cancer vaccines with CAR‐T cell therapy

Traditional vaccines played an essential role in combating immune‐related diseases and saving countless lives of patients with infectious diseases [175]. Researchers are now developing cancer vaccines to treat various cancers. These cancer vaccines include preventive vaccines and therapeutic vaccines. Preventive vaccines protect the immunized person from specific tumors by inducing the immunized person to form immune memories to specific cancers [176]. Therapeutic cancer vaccines could evoke the anti‐cancer function of the body by enhancing immunogenicity, increasing the activity of endogenous T cells and other mechanisms. Therapeutic cancer vaccines are mainly divided into three categories: cell vaccine, protein/polypeptide vaccine and nucleic acid vaccine [177, 178, 179].

Recently, the combined therapeutic effect of CAR‐T cells and cancer vaccines has been continuously tested in multiple cancer types. Reinhard *et al*. found that the CAR‐T cells created with tight junction protein claudin 6 (CLDN6) therapy in combination with the RNA‐lipoplex (RNA‐LPX) vaccine encoding CLDN6 could promote CLDN6‐CAR‐T cell proliferation. It can effectively eliminate human ovarian and mouse lung cancer cells in mouse models, which has been verified in both mouse and human cancer cells. The CLDN18.2‐LPX vaccine combined with CLDN18.2‐CAR‐T cell therapy, consistently achieved a similar anti‐tumor effect when compared with CLDN6‐CAR‐T cell therapy [180]. It was reported that the dendritic cells (DC) loaded with the same antigens with CAR‐T cells can be used as DC vaccine to enhance the activation, expansion and persistence of CAR‐T cells and improve the anti‐tumor effect on K‐562 (human chronic myeloid leukemia cell) cancer cells [181, 182]. Ma *et al*. also designed a vaccine containing an amphiphilic CAR‐T ligand (amph‐ligand) by connecting the vaccine with the fat molecule lipid tail, which can be recognized and combined with albumin in the blood and directly transported to lymph nodes. When these kinds of vaccines enter lymph nodes, they can significantly improve T cell activity, trigger the expansion of CAR‐T cells and ultimately increase the therapeutic effect. The strategy could effectively prevent tumor recurrence, form long‐term immune memory, and improve the anti‐tumor effect of CAR‐T cells [183, 184].

In hematological malignancies, Cheloni *et al*. found that the tumor cells fused with dendritic cells from patients could be used as DC/tumor vaccine to improve significantly the durability of CAR‐T cells [185]. Wang *et al*. generated a CD19‐CAR variant using cytomegalovirus (CMV)‐specific T cells. The CAR‐T cells constructed with CMV‐specific T cells may be sensitive to CMV vaccine, which may stimulate the persistence and proliferation of CAR‐T cells *in vivo*. Furthermore, this strategy was also found to improve the anti‐tumor ability measured in the lymphoma tumor‐bearing mouse models [186]. Altogether, in recent years, the combination of vaccines and CAR‐T cell therapy in treating cancers has seen some success and has prompted the evaluation of this approach in clinical trials (Table 2).

**Table 2:** The combined therapies of CAR‐T cells and vaccine in preclinical trial. The clinical trial data were collected from ClinicalTrials.gov using the keyword Vaccine CAR‐T (https://clinicaltrials.gov/ct2).

Study title	Conditions/treatments	Phase	NCT number	Sponsor
Genetically modified T‐cells (CMV‐Specific CD19‐CAR T cells) plus a vaccine (CMV‐MVA Triplex) for the treatment of intermediate or high grade B‐cell non‐Hodgkin's lymphoma	Intermediate/high Grade B‐cell non‐Hodgkin's lymphoma Recurrent B‐Cell non‐Hodgkin's lymphoma Refractory B‐Cell non‐Hodgkin's lymphoma	I	NCT05801913	City of Hope Medical Center, Duarte, CA, USA
Optimizing cellular and humoral immunity by vaccinating with PCV13 before and after CAR‐T therapy	Diffuse large‐cell lymphoma primary mediastinal large B‐cell lymphoma (PMBCL) Transformed follicular lymphoma (TFL) High‐grade B‐cell lymphoma (HGBCL) Follicular lymphoma	II	NCT04745559	Moffitt Cancer Center, Tampa, FL, USA
Genetically modified T‐cells (CMV‐Specific CD19‐CAR T‐cells) plus a vaccine (CMV‐MVA Triplex) following stem cell therapy for the treatment of intermediate or high grade B‐cell non‐Hodgkin's lymphoma	B‐cell non‐Hodgkin's lymphoma Diffuse large B‐cell lymphoma Mantle cell lymphoma Recurrent B‐cell non‐Hodgkin's lymphoma Recurrent diffuse large B‐cell lymphoma Recurrent Mantle cell lymphoma Recurrent transformed non‐Hodgkin's lymphoma Transformed non‐Hodgkin's lymphoma	I	NCT05432635	City of Hope Medical Center, Duarte, CA, USA
NGS‐MRD assessment of combination immunotherapies targeting T‐ALL	T cell acute lymphoblastic leukemia	I	NCT05277753	Shenzhen Geno‐immune Medical Institute Shenzhen, Guangdong, China
NGS‐MRD assessment of combination immunotherapies targeting B‐ALL	B‐cell acute lymphoblastic leukemia	I	NCT05262673	Shenzhen Geno‐Immune Medical Institute Shenzhen, Guangdong, China

### 12.4 Nano‐material‐based therapy and CAR‐T cell therapy

Nano‐materials are characterized by their small size, large surface area and volume ratio, and good thermal and mechanical catalytic properties [187, 188]. In recent years, nano‐materials have developed rapidly, and nano‐particles can effectively deliver drugs to the tumor site and release the drugs in controlled speed and amount, thus improving drug safety and therapeutic effect [189]. Moreover, nano‐particles play a critical role in the diagnosis, detection and imaging of diseases [190, 191, 192]. With the development of nanotechnology, nano‐materials are now widely studied and have been combined with other methods to treat diseases in a more efficient manner [193, 194].

Due to the specific characteristics of nano‐materials, the combination of CAR‐T cell therapy with nanotechnology was hypothesized to reduce or eliminate some limitations and side effects of CAR‐T cell therapy in treating solid tumors [195]. The generation of traditional CAR‐T cells is a relatively time‐consuming process of about 6–14 days. Initially, T cells were isolated and purified from cancer patients. The cells then had to be transformed and ectopically expressed the CAR genes by viral vector transduction and non‐viral vector transfection. Ultimately, the CAR‐T cells had to be expanded to a large quantity *in vitro* and then injected into patients to exert their anti‐tumor function [196, 197]. Interestingly, nano‐materials could deliver CAR plasmids into the body and directly edit T cells to produce CAR‐T cells *in vivo*. Smith *et al*. used nanoparticles to carry a plasmid capable of producing CAR‐T cells *in vivo* to be anchored on the surface of T cells by combining with specific targets of T cells to form CD19‐targeted CAR‐T cells *in vivo*. This approach showed a therapeutic activity equivalent to that of traditional CAR‐T cells in leukemia treatment [194]. Recently, researchers have developed the CD3 antibody modified LNP, which are loaded with the plasmids for IL‐6 shRNA and CD19‐CAR (anti‐CD3‐LNP/CAR19 + shIL‐6). The modified LNP can stably transform T cells into *IL‐6* knockout CAR‐T cells, kill the tumor cells that highly express CD19, and further reduce the release of IL‐6 and CRS in leukemia *in vivo* [198]. Researchers also used CD5‐loaded LNP to deliver the modified CAR mRNA into T lymphocytes, which instantly produced effective CAR‐T cells.

The tumor microenvironment (TME) plays a vital role in regulating the effect of immunotherapy. An immunosuppressive TME is also a major factor hampering the function of CAR‐T cells partially by restricting CAR‐T cells from reaching the tumor site to bind with the corresponding antigen and exert an anti‐tumor function [199]. Notably, nanotechnology may have the capacity to help CAR‐T cells break the barrier due to an immunosuppressive TME. Zhang *et al*. designed tumor‐targeting peptide iRGD (CRGDK/RGPD/EC) lipid nanoparticles loaded with phosphatidylinositol‐3 kinase (PI3K) inhibitors and α‐galactosylceramide (therapeutic T cell agonist), which can not only stimulate T cell function but also inhibit immune‐suppressive tumor cells. Notably, the CAR‐T cells can efficiently approach and penetrate the breast tumor more effectively to havey a better anti‐tumor function in the breast cancer model [200]. In addition, Chen *et al*. developed indocyanine green nanoparticle (INP)‐based biohybrid CAR‐T (CT‐INP) cells, which could serve as a potential nano‐photosensitizer. When combined with irradiation, INP can generate heat from irradiation and damage the extracellular matrix but not the cells, reduce the dense structure of solid tumors, release antigens and improve the accumulation and anti‐tumor function of CAR‐T cells [201, 202]. A nano‐enzyme with dual properties of photothermal‐nanocatalysis could break the dense structure of the TME in a non‐small cell lung cancer (NSCLC) mouse model, and exert an improved anti‐tumor activity combined with B7‐H3‐CAR‐T cell therapy [203]. It is well known that the A2a adenosine receptor (A2aR) is expressed on T cells and can be activated by adenosine (immunosuppressive factor) and restrict the function of CAR‐T cells [204]. In line with this notion, Siriwon *et al*. [205] used cross‐linked multilamellar liposomal vesicles (cMLV) to deliver specific A2aR antagonist (SCH‐58261) to TME and enhanced the anti‐tumor effect in an ovarian cancer‐bearing mouse model by reactivating the tumor‐infiltrating lymphocytes (TIL) inactivated by immunosuppressive molecules such as adenosine.

To improve further the therapeutic effect of CAR‐T cells, immune activating factors (such as IL‐2 and IL‐15) are commonly used to augment safely the function of effective T cells and NK cells during immunotherapy. Researchers designed a nanogel (NG) coated with immune activating factors such as the IL‐15 super agonist complex to deliver selectively expanded T cells into the melanin tumor site. They found that, compared with free immune activating factor, NG achieved a 16‐fold increase in T cell delivery to tumors and an eight‐fold dose administration without toxicity which significantly improved the therapeutic effect of CAR‐T cells [206]. Immune checkpoint molecules CTLA‐4 and PD‐L1 can inhibit the activation and function of immune T cells [207]. Although immune checkpoint inhibitors (ICIs) can block CTLA‐4 or PD‐L1 to reactivate T cells and induce tumor killing, they also cause systemic toxicity and have some adverse immune effects [208]. In addition, some immunomodulators and cytokines (IL‐12) can also be delivered by nano‐materials in the CAR‐T cell therapy to enhance the anti‐tumor role of CAR‐T cells [198, 209].

Although nanotechnology can overcome some limitations of CAR‐T cell therapy and has achieved satisfactory outcomes in preclinical research, many problems still need to be solved before it is used in clinical practice. Applying nanotechnology in CAR‐T cell therapy might be a promising therapeutic strategy in combating tumors in the near future.

The combination of radiotherapy, chemotherapy, vaccines and nanomedicine with CAR‐T cell therapy could significantly improve the therapeutic effect of CAR‐T cells. In addition, the oncolytic virus (OV) could enhance the therapeutic effect of CAR‐T cells by promoting T cell initiation and infiltration [210]. Furthermore, different immunotherapies could be combined to achieve better treatment outcomes. For example, the blockage of immune checkpoints such as PD‐1 (programmed cell death 1) and PD‐L1 (programmed cell death ligand 1) can further significantly improve the therapeutic effect of CAR‐T cells [211, 212]. The combination of other therapeutic strategies with CAR‐T cell therapy might thus be a promising therapeutic regimen for the treatment of malignant tumors.

## 13 Conclusions and outlook

At present, cancer is one of the malignant diseases that threaten human health worldwide. Among the tumor treatment strategies, CAR‐T cell therapy is a newly developed immunotherapy that has achieved remarkable results in treating some hematological malignancies. However, some obstacles need to be overcome before CAR‐T cell therapy can be successfully applied to treat solid tumors. These obstacles include antigen escape, tumor infiltration, immune suppressive tumor microenvironment and the migration of CAR‐T cells. Novel strategies are being explored to generate new CAR‐T cells to increase their therapeutic effect.

Notably, researchers have improved the infiltration ability and therapeutic effect of CAR‐T by optimizing the delivery of CAR genes and the administration regimen of CAR‐T cells. CAR‐T cells were designed to target multiple tumor antigens or other molecules simultaneously to solve the problem of antigen escape. In addition, ‘AND’, ‘OR’ and ‘NOT’ logic gate controls were included in the design of new CAR‐T to better activate CAR‐T cells and effectively overcome antigen escape and reduce non‐targeted toxicity on normal cells. In addition, super CAR‐T cells use ZipCAR and ZipFv to form a separate, universal programmable CAR‐T system that improves the safety and flexibility of CAR‐T cells and is more conducive to tumor resistance. The researchers have also used molecular switches to regulate CAR‐T cells remotely, such as adding photothermal switches to design small molecular compounds. Recently, SNIPCAR was designed to improve the flexibility and effectiveness of CAR‐T cells. To overcome the time‐consuming preparation of autologous CAR‐T cells and the difficulty of preparing these for special individuals, researchers designed allogeneic CAR‐T cells and measures to solve the problems, such as GVHD and HVGR using gene editing. In addition, specific targets are of great significance for CAR‐T cells, so researchers have discovered some new findings on targets, such as ALPL‐1 for metastatic osteosarcoma and GPRC5D for MM, which provide a better choice of targets for accurate treatment of tumors. The CAR‐T cells produced by nano‐antibodies also show some advantages; nano‐antibodies are easier to humanize and, because of their unique structure, are not easy to aggregate and lead to the depletion of CAR‐T cells, so they can play a better role in double‐target therapy. In addition, the use of gene‐editing technology improves the transmission of CAR gene and also plays a role in improving transport and persistence.

In addition, the combined use of CAR‐T cells and other methods has also been improved. Bridging chemotherapy and gonorrhea treatment provide a better and more suitable *in vivo* environment for the treatment of CAR‐T cells. Radiotherapy can improve the therapeutic effect of CAR‐T cells by inhibiting the growth of tumor cells, increasing the anti‐tumor function of immune cells and improving the tumor microenvironment infiltration of CAR‐T cells. The combination of tumor vaccine and CAR‐T can trigger the expansion of CAR‐T cells, prevent tumor recurrence and form immune memory. In addition, the delivery of nano‐materials and the synthesis of CAR‐T cells *in vivo* and the transmission of some cytokines were also used to improve the therapeutic effect of CAR‐T cells. Importantly, combination therapy of different therapeutic methods could achieve better treatment effects or reverse the drug resistance frequently found during cancer treatment. Therefore, the novel therapeutic strategy in combination with CAR‐T cell therapy was thoroughly investigated.

Altogether, although CAR‐T cell therapy has been extensively studied in the treatment of cancer and developed rapidly, there is still a long way to go to reduce the treatment cost, effectively treat solid tumors and reduce the side effects of CAR‐T cell therapy; this warrants further investigation for the development of novel CAR‐T cell therapeutic methods.

## Author contributions

MW and LJ wrote and revised the paper. XZ and XD edited and revised the paper. All authors approved the final article.
